# CEA but not CA19-9 is an independent prognostic factor in patients undergoing resection of cholangiocarcinoma

**DOI:** 10.1038/s41598-017-17175-7

**Published:** 2017-12-05

**Authors:** Sven H. Loosen, Christoph Roderburg, Katja L. Kauertz, Alexander Koch, Mihael Vucur, Anne T. Schneider, Marcel Binnebösel, Tom F. Ulmer, Georg Lurje, Wenzel Schoening, Frank Tacke, Christian Trautwein, Thomas Longerich, Cornelis H. Dejong, Ulf P. Neumann, Tom Luedde

**Affiliations:** 1 Department of Medicine III, University Hospital RWTH Aachen, Pauwelsstrasse 30, 52074 Aachen, Germany; 2 Division of Gastroenterology, Hepatology and Hepatobiliary Oncology, University Hospital RWTH Aachen, Pauwelsstrasse 30, 52074 Aachen, Germany; 3 Department of Visceral and Transplantation Surgery, University Hospital RWTH Aachen, Pauwelsstrasse 30, 52074 Aachen, Germany; 4 Institute of Pathology, University Hospital RWTH Aachen, Pauwelsstrasse 30, 52074 Aachen, Germany; 5 Department of Surgery, Maastricht University Medical Centre (MUMC), PO Box 5800 Maastricht, The Netherlands

## Abstract

Cholangiocarcinoma (CCA) represents a rare form of primary liver cancer with increasing incidence but dismal prognosis. Surgical treatment has remained the only potentially curative treatment option, but it remains unclear which patients benefit most from liver surgery, highlighting the need for new preoperative stratification strategies. In clinical routine, CA19-9 represents the most widely used tumor marker in CCA patients. However, data on the prognostic value of CA19-9 in CCA patients are limited and often inconclusive, mostly due to small cohort sizes. Here, we investigated the prognostic value of CA19-9 in comparison with other standard laboratory markers in a large cohort of CCA patients that underwent tumor resection. Of note, while CA19-9 and CEA were able to discriminate between CCA and healthy controls, CEA showed a higher accuracy for the differentiation between CCA and patients with primary sclerosing cholangitis (PSC) compared to CA19-9. Furthermore, patients with elevated levels of C-reactive protein (CRP), CA19-9 or CEA showed a significantly impaired survival in Kaplan-Meier curve analysis, but surprisingly, only CEA but not CA19-9 represented an independent predictor of survival in multivariate Cox-regression analysis. Our data suggest that CEA might help to identify CCA patients with an unfavourable prognosis after tumor resection.

## Introduction

Although cholangiocarcinoma (CCA) represents the second most common primary hepatic malignancy, it can be considered a rare type of gastrointestinal cancer comprising less than 1% of all human malignancies^1,2^. Depending on the geographical region, incidence rates vary from 0.5 to 3.4 cases per 100.000/year in the US and Western Europe to 85 cases per 100.000 people in some parts of Asia^3^. With respect to its anatomical localization, CCA is subdivided into intrahepatic and extrahepatic CCA, the latter comprising perihiliar CCA (Klatskin tumor) and distal CCA^4^. Systemic chemotherapy with cisplatin and gemcitabine is the standard treatment for locally-advanced and metastatic disease^2^, while for early stage CCA, surgical resection represents a potentially curative treatment. However, even after resection, the patients’ prognoses often remain limited. As such, following R_0_ resection, 5-year survival rates vary between 23–37%^5–8^. Importantly, beyond the standard diagnostic criteria that define if a patient is resectable or not (e.g. tumor stage, presence of metastases, clinical performance status, liver function etc.)^9–12^, it is still not fully clear which patients actually benefit from extensive liver surgery in terms of post-operative survival and which patients continue to have a poor prognosis even after tumor resection. Therefore, novel stratification strategies including easily accessible parameters are urgently needed to determine the ideal therapeutic approach for individual CCA patients.

As disease-specific biomarkers for CCA have not been established to date^2^, carbohydrate antigen 19–9 (CA19-9), the standard tumor marker for pancreatic adenocarcinoma^13,14^, is the most frequently used biomarker for diagnostic and treatment predictive purposes in CCA patients in clinical practice^15,16^. Besides, CA19-9 has also been suggested as a prognostic marker for CCA patients undergoing tumor resection^17,18^ and patients receiving chemotherapy^19^. Nevertheless, serum levels of CA19-9 are also elevated in patients with non-malignant biliary diseases such as primary sclerosing cholangitis or biliary obstruction due to choledocholithiasis^20,21^, implying that CA19-9 might not be the ideal tool for surveillance of patients with benign biliary disease. Carcinoembryonic antigen (CEA), a well-established tumor marker particularly in the field of colorectal and other adenocarcinomas^22,23^, has gained increasing attention as a potential biomarker for hepatobiliary malignancies^24^. Similarly, C-reactive protein (CRP) was recently suggested to be an independent prognostic factor for patients with intrahepatic^25^ and perihiliar CCA^26^. However, given the small sample size of most studies in CCA patients^15,27–29^, the diagnostic and prognostic values of these respective markers specifically in the clinical setting of surgical resection have remained inconclusive. Here, we therefore aimed to evaluate the diagnostic and prognostic capabilities of CA19-9, CEA, CRP and other routinely measured laboratory parameters in a large cohort of CCA patients undergoing tumor resection at our tertiary referral centre between 2010 and 2016 in comparison to healthy control samples and patients with primary sclerosing cholangitis.

## Results

### Patients characteristics

All 190 patients CCA patients that were included into this study underwent surgical tumor resection at the Department of Surgery at University Hospital RWTH Aachen, a tertiary referral hospital. The median age of the study population was 68 years. 78 patients (40.8%) were treated for intrahepatic CCA, 81 patients (42.6%) for Klatskin tumors, 16 patients (8.4%) for distal CCA and 15 patients (7.9%) for gallbladder carcinoma. Regarding the T-stage, 10.5% of patients suffered from T1-, 43.2% from T2-, 30.9% from T3- and 15.4% of T4-stage. 46.8% of the included patients had node-negative disease (N0), while 53.2% of patients showed a tumor spreading into lymph nodes (N1). 18.1% of patients had extrahepatic CCA with intrahepatic or local peritoneal tumor spreading (M1). The tumours of 33.6% of patients were graded as poorly differentiated (G3) and 66.4% of patients showed a moderately differentiated tumor. 52% of the CCA study population died during the follow-up period. The median overall survival was 703 days. Patient characteristics are summarized in Table 1.

**Table 1:** Characteristics of study population.

	Study population
CCA patients	190
PSC patients	24
Healthy controls	50
Sex [%]:	
male-female (CCA)	59.5–40.5
male-female (PSC)	70.8–29.2
Age [years, median and range]	
CCA	68 [35–84]
PSC	41 [20–51]
BMI [kg/m^2^, median and range]	
CCA	25.61 [18.83–46.36]
Anatomic location of CCA [%]	
Intrahepatic	40.7
Klatskin	42.9
Distal	8.5
Gallbladder	7.9
Staging and grading of CCA [%]	
T1-T2-T3-T4	10.5–43.2–30.9–15.4
N0-N1	46.8–53.2
M0-M1	81.9–18.1
G2-G3	66.4–33.6
R0-R1	67.1–32.9
Death during follow up [%]	
Yes-No	52–48

### Serum levels of CA19-9 and CEA are elevated in patients with CCA

**Figure 1:**
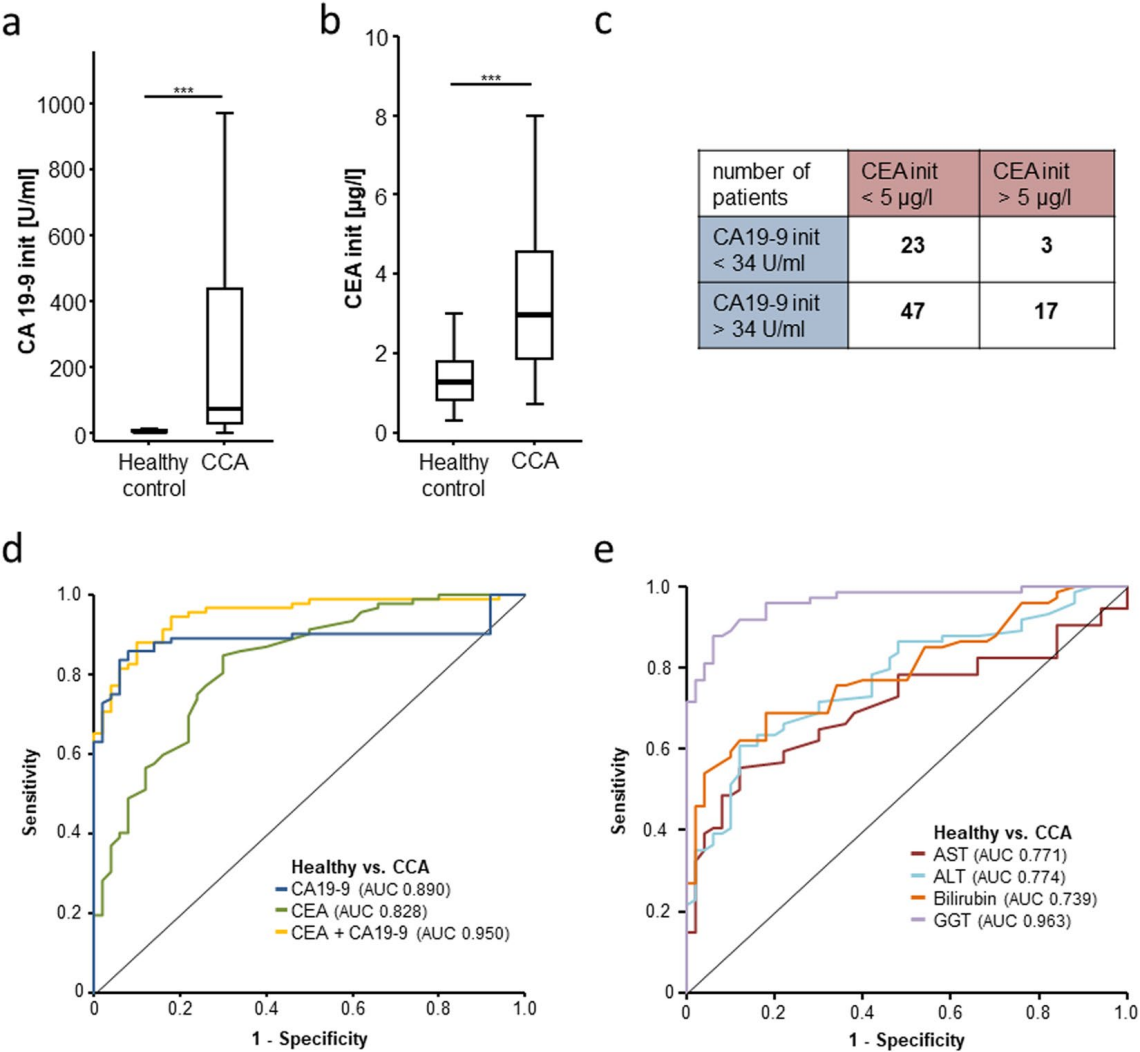
Serum levels of CA19-9 and CEA are elevated in patients with CCA. Initial serum levels of CA19-9 (**a**) and CEA (**b**) are significantly elevated in patients with CCA compared to healthy controls. 47 patients (52.2%) showed an isolated elevation of CA19-9, 3 patients (3.3%) an isolated elevation of CEA and 17 patients (18.9%) a combined elevation of both markers, while 23 patients (25.6%) did not display elevated levels of tumor markers when applying our laboratory’s standard cut-off values (**c**). ROC curve analysis reveals and AUC values of 0.890 and 0.828 for CA19-9 and CEA for the differentiation between CCA and healthy controls (**d**). Of routinely tested serum markers for liver injury, only GGT displays a superior AUC when analysed in this context (**e**).

In our cohort of 190 patients, preoperative CA19-9 and CEA levels were retrospectively available in 90 patients (Table 2). Compared to healthy controls (n = 50), both tumor markers were significantly elevated in individuals with CCA (Fig. 1a,b, Table 2). Similarly, serum markers of liver injury and biliary obstruction (aspartate transaminase (AST), alanine transaminase (ALT), bilirubin, gamma-glutamyltransferase (GGT) and alkaline phosphatase (ALP)) and inflammation (CRP) were also elevated above the clinically established cut-off values (upper limit of normal), whereas the median leucocyte count of CCA patients was within the normal range (Table 2). Of the 90 patients with available tumor marker data, 47 patients (52.2%) showed an isolated elevation of CA19-9, 3 patients (3.3%) an isolated elevation of CEA and 17 patients (18.9%) showed an elevation of both markers, while 23 patients (25.6%) did not display elevated levels of tumor markers when applying our laboratory’s standard cut-off values (CEA: 5 µg/l, CA19-9: 34 U/ml) (Fig. 1c). To determine the diagnostic accuracy of these tumor markers, we performed ROC curve analysis revealing AUC values of 0.890 and 0.828 for CA19-9 and CEA for the differentiation between CCA and healthy controls, respectively (Fig. 1d). The combination of CA19-9 and CEA was superior to either marker alone, showing an AUC of 0.950 for the differentiation between CCA and healthy controls (Fig. 1d). Notably, of the routinely tested markers, only GGT displayed a superior AUC when analysed in this context (AUC_AST_ 0.771, AUC_ALT_ 0.774, AUC_bilirubin_ 0.739, AUC_GGT_ 0.943; Fig. 1e). Optimal diagnostic cut-off values were established using the Youden-index method. For CEA, a serum level of 1.55 µg/l differentiated best between CCA and healthy controls showing a sensitivity and specificity of 85 and 70%, respectively. CA19-9 serum levels displayed an optimal diagnostic power with a sensitivity of 85.9% and a specificity of 92% at a cut-off value of 15.75 U/ml. Interestingly, both diagnostic cut-off values in our specific cohort of CCA patients were clearly lower than the standard cut-off values used in daily routine.

**Table 2:** Serum Levels of laboratory markers.

	CCA patients median [range], number of analyzed patients	PSC patients median [range], number of analyzed patients	Healthy controls median [range], number of analyzed patients
CEA [µg/l]	2.95 [0.71–110.4], n = 92	1.15 [0.2–5.1], n = 20	1.25 [0.3–6.3], n = 50
CA 19–9 [U/ml]	78.4 [0.5–38092], n = 99	15.95 [2.3–78.4], n = 24	5.4 [0–44.1], n = 50
AFP [ng/ml]	4 [1–177.8], n = 49	1.95 [1–8.7], n = 20	—
WBC [cells/nl]	7.95 [2.9–21.8], n = 190	6.8 [3.3–12.4], n = 23	—
CRP [mg/l]	14.95 [0–230], n = 180	6.4 [0.6–127], n = 20	—
AST [U/l]	45 [15–1587], n = 189	67 [16–108], n = 22	28 [20–78], n = 50
ALT [U/l]	46.5 [10–1097], n = 136	75.5 [22–224], n = 24	20 [5–82], n = 50
GGT [U/l]	293.5 [13–2015], n = 184	265 [18–1223], n = 24	17 [8–120], n = 50
ALP [U/l]	215 [45–1655], n = 184	248 [81–694], n = 24	65 [36–102], n = 50
Bilirubin [mg/dl]	0.9 [0.2–21.49], n = 189	0.89 [0.36–11.07], n = 22	0.41 [0.1–1.46], n = 50
Creatinine [mg/dl]	0.8 [0.4–1.9], n = 190	0.78 [0.3–2.42], n = 23	—
Sodium [mmol/l]	140 [121–146], n = 189	139 [136–144], n = 23	—
Potassium [mmol/l]	4.3 [2.9–7], n = 189	4.25 [3.6–5.3], n = 22	—
Calcium [mmol/l]	2.3 [1.78–5.43], n = 182	2.35 [2.09–2.52], n = 21	—
Haemoglobin [g/l]	12.55 [7.8–19.9], n = 190	14.4 [8.3–16.5], n = 23	—
Platelets [cells/nl]	276.5 [9–931], n = 190	261 [118–417], n = 23	—

WBC: white blood cell count, CRP: C-reactive protein, AST: aspartate transaminase, ALT: alanine transaminase, GGT: γ-Glutamyl transpeptidase, ALP: alkaline phosphatase, CEA: carcinoembryonic antigen, CA 19-9: carbohydrate-Antigen 19-9, AFP: Alpha-fetoprotein.

We also analysed if serum levels of the tumor markers (CA19-9 and CEA) or inflammatory markers (CRP and leucocyte count) were altered between patients with different T-, N- or M-status (according to UICC TNM classification, 7^th^ edition 2010^30^). While circulating levels of CA19-9 and CEA as well as the leucocyte count were unaltered between different T-stages (Suppl. Figure 1a, b and d), patients with a more advanced local tumor stage (T2, T3 and T4) had significantly higher CRP levels compared to T1-stage patients (Suppl. Figure 1c). Similarly, CRP levels were elevated in patients with node-positive disease (N1, Suppl. Figure 1g) whereas no difference for CA19-9, CEA and the leucocyte count became apparent (Suppl. Figure 1e,f and h). CCA patients with extrahepatic CCA and intrahepatic or local peritoneal tumor spreading (M1) that were still eligible for tumor resection showed a non-significant trend towards higher levels of CA19-9 and CEA (Suppl. Figure 1i and j) and displayed significantly elevated levels of CRP and the leucocyte count (Suppl. Figure 1k and l). When comparing the different anatomical tumor localizations (intrahepatic CCA, Klatskin tumors, distal CCA and gallbladder cancer) we observed no significant differences regarding CA19-9, CEA and CRP levels as well as the leucocyte count (Suppl. Figure 2a–d). However, patients with gallbladder carcinoma showed a non-significant trend towards higher levels of CA19-9 and CEA compared to other localizations (Suppl. Figure 2a and b). Interestingly, poorly differentiated tumours (G3) showed a strong but non-significant trend (p = 0.066) towards higher levels of CEA compared to moderately differentiated tumours (G2) (Suppl. Figure 2f), whereas serum levels of CA19-9, CRP and the leucocyte count were unaltered between G2 and G3 tumours (Suppl. Figure 2e, g and h). Finally, we found that the subgroup of patients that were resected with positive resection margins (R1) had elevated initial CA19-9 and CRP but not CEA serum levels or elevated leucocyte count (Suppl. Figure 2i-l).

### Serum levels of CA19-9 and CEA in patients with primary sclerosing cholangitis

**Figure 2:**
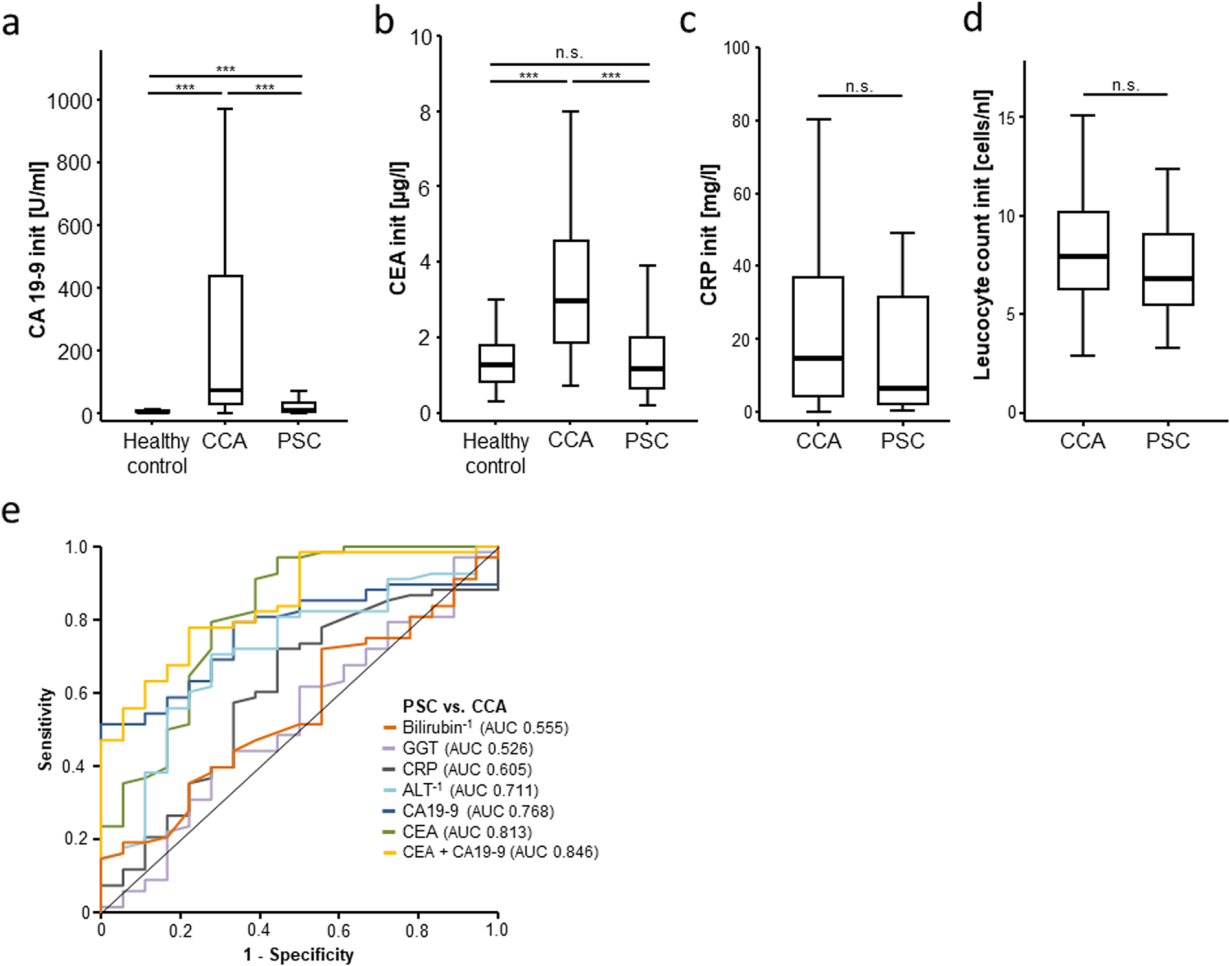
Serum levels of CA19-9 and CEA in patients with primary sclerosing cholangitis. CA19-9 (**a**) and CEA (**b**) serum levels are significantly elevated in CCA patients not only in comparison to healthy controls (as shown in Fig. 1) but also in comparison to PSC patients. CA19-9 (**a**) but not CEA (**b**) serum levels are also elevated in PSC patients compared to healthy controls. Serum levels of CRP (**c**) and the leucocyte count (**d**) were unaltered between CCA and PSC patients. ROC curve analysis shows that serum levels of CEA are superior to CA19-9 levels in differentiating between patients with PSC and CCA (**e**). Standard liver serum markers (ALT, bilirubin, GGT) as well as CRP serum levels are unsuitable for the differentiation between PSC and CCA patients (**e**).

As several studies suggested that CA19-9 and CEA are also elevated in the serum of patients with benign cholangiopathies and might therefore be unsuitable for early detection of CCA in this setting^15,31^, we next compared serum levels of CA19-9 and CEA in patients with CCA and patients with primary sclerosing cholangitis (PSC, n = 24) and no evidence of malignant transformation. Both CA19-9 and CEA serum levels were significantly higher in CCA patients compared to PSC patients (Fig. 2a,b). Importantly, PSC patients displayed only elevated CA19-9 levels (Fig. 2b) but not CEA levels (Fig. 2a), corroborating previous findings of elevated CA19-9 levels in non-malignant bile duct diseases^31^. Serum levels of CRP and the leucocyte count were unaltered between CCA and PSC patients (Fig. 2c,d). In line with this finding, ROC curve analysis revealed that serum levels of CEA (AUC: 0.813) were superior to CA19-9 levels (AUC: 0.768) in differentiating between patients with PSC and CCA (Fig. 2e). Again, we used Youden-index method and determined an optimal cut-off value of 1.85 µg/l and 78.9 U/ml for CEA and CA19-9 respectively for the discrimination between PSC and CCA. The best diagnostic power for the differentiation between PSC and CCA patients was achieved when combining CEA and CA19-9 showing an AUC of 0.846 (Fig. 2e). Importantly, these findings remained unchanged when PSC patients were compared to intrahepatic CCA and Klatskin tumor patients only, which represent the most frequent tumor entities occurring in PSC patients (data not shown). In contrast, the standard liver serum markers (ALT, bilirubin, GGT) as well as CRP serum levels were unsuitable for the differentiation between PSC and CCA patients (Fig. 2e). Together this exploratory analysis suggests that in a setting of PSC, the clinical implication of a moderate CA19-9 elevation is often difficult to interpret, but even subtle elevations of CEA could be a serious warning sign for malignant transformation. However, this hypothesis would warrant further confirmation, ideally in a larger and prospective cohort of PSC patients.

### Preoperative CEA but not CA19-9 or CRP serum levels are an independent predictor of long-term mortality after CCA tumor resection

Based on previous studies suggesting a prognostic function of CA19-9 in patients with cholangiocarcinoma^18,32–35^, we next evaluated the prognostic value of CA19-9, CEA, CRP and the leucocyte count in our cohort of patients. For this, we first performed ROC curve analyses which revealed AUC values of 0.649 (CA19-9), 0.602 (CEA), 0.666 (CRP) and 0.534 (leucocyte count) for the differentiation between patient that died during the follow-up period and patients that were still alive (Suppl. Figure 3a and b). We next performed Kaplan-Meier curve analysis by subdividing our cohort of patients for each serum marker into two groups with respective serum levels above or below the 50^th^ percentile of our cohort. Using these respective cut-off levels, only CA19-9 and CRP but not CEA and the leucocyte showed a significantly impaired long-term survival in patients with serum levels above the respective 50^th^ percentile (Suppl. Figure 3c-f). Off note, serum levels of alpha-fetoprotein (AFP), a widely used biomarker for hepatocellular carcinoma^36^, were unsuitable for the prediction of long term survival in CCA patients (Suppl. Figure 3g).

As the median level of the respective serum markers might not represent the ideal cut-off value for the differentiation between survivors and non-survivors in our cohort, we next used the Youden-index method to establish ideal cut-off values for each serum marker.

**Figure 3:**
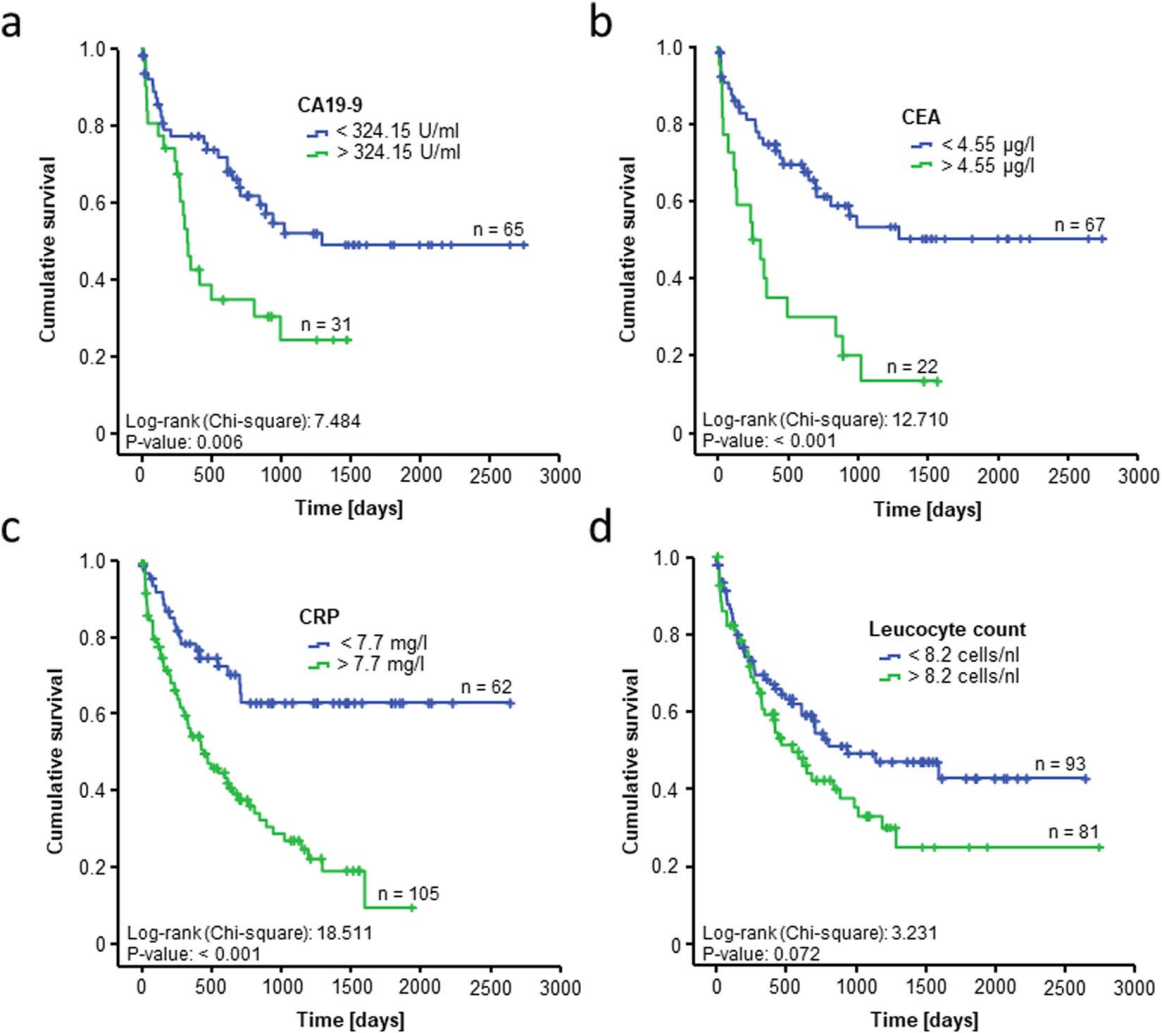
Evaluation of CEA, CA19-9, CRP and leucocyte count as prognostic marker for CCA patients after tumor resection. Using our optimal prognostic cut-off value, Kaplan-Meier curve analysis shows highly significant impaired long-term survival for patients with a CA19-9 serum level above 324.15 U/ml (**a**), a CEA serum level above 4.55 µg/l (**b**) and a CRP level above 7.7 mg/L (**c**), respectively. The ideal cut-off value for the leucocyte count of 8.2 cells/nl is unable to discriminate long-term survivors and non-survivors (**d**).

The optimal cut-off values for CA19-9 and CRP were 324.15 U/ml and 7.7 mg/L, respectively which further increased the prognostic potential of these markers (Fig. 3a,c). Interestingly, Kaplan-Meier curve analysis using the optimal CEA cut-off value of 4.55 µg/l revealed an highly significant impaired long-term survival for patients with CEA serum levels above 4.55 µg/l (Fig. 3b). In contrast, the ideal cut-off value for the leucocyte count of 8.2 cells/nl was not able to significantly discriminate between patients that succumbed to death early and patients with long-term survival (Fig. 3d).

To further substantiate the prognostic power of the investigated parameters, we next performed univariate Cox-regression analyses of routinely measured tumor markers for hepatobiliary malignancies (CA19-9, CEA and AFP) and standard laboratory markers (haemoglobin, leucocyte count, thrombocyte count, sodium, potassium, calcium, AST, ALT, bilirubin, GGT, AP, and CRP) as well as routine clinical and pathological parameters (age, gender, body mass index (BMI) and TNM-stages). In this univariate analysis, serum levels of CEA, CRP, sodium, haemoglobin and ALP as well as the TNM stage but not CA19-9 or the leucocyte count were predictive factors for long-term survival (Table 3). Subsequently, we included those parameters with a significant result in the univariate analysis into a multivariate Cox-regression analysis. Importantly, this multivariate analysis revealed that besides the T- and M-status, preoperative CEA levels represented the only serum marker that predicted long-term survival after CCA tumor resection (Table 3).

**Table 3:** Univariate and multivariate Cox-regression analyses of different laboratory and clinical markers for the prediction of long-term survival.

Parameter	Univariate Cox-regression		Multivariate Cox-regression	
	p-value	Hazard-Ratio (95% CI)	p-value	Hazard-Ratio (95% CI)
CEA	<0.001***	1.046 (1.026–1.067)	0.035*	1.030 (1.002–1.058)
CA19-9	0.075	1.000 (1.000–1.000)		
AFP	0.287	0.966 (0.907–1.029)		
CRP	<0.001***	1.010 (1.006–1.014)	0.219	1.008 (0.995–1.021)
WBC	0.064	1.059 (0.997–1.126)		
Sodium	0.026*	0.950 (0.907–0.994)	0.872	1.011 (0.882–1.159)
Potassium	0.797	0.942 (0.597–1.487)		
Calcium	0.381	1.345 (0.693–2.612)		
Haemoglobin	0.009**	0.825 (0.715–0.953)	0.475	0.896 (0.663–1.211)
Thrombocytes	0.763	1.000 (0.998–1.001)		
AST	0.934	1.000 (0.999–1.001)		
ALT	0.624	0.999 (0.997–1.002)		
Bilirubin	0.269	1.030 (0.977–1.085)		
GGT	0.073	1.000 (1.000–1.001)		
ALP	0.048*	1.001 (1.000–1.002)	0.947	1.000 (0.997–1.003)
Creatinine	0.693	1.169 (0.539–2.533)		
Age	0.929	0.999 (0.979–1.020)		
BMI	0.198	1.030 (0.958–1.077)		
Sex	0.395	1.203 (0.786–1.843)		
T stage (T_1/2_ vs. T_3/4_)	0.002**	2.042 (1.290–3.230)	0.047*	2.742 (1.015–7.404)
N stage	<0.001***	2.651 (1.628–4.317)	0.769	1.177 (0.396–3.501)
M stage	<0.001***	2.963 (1.801–4.876)	0.009*	4.865 (1.472–16.075)

WBC: white blood cell count, CRP: C-reactive protein, AST: aspartate transaminase, ALT: alanine transaminase, GGT: γ-Glutamyl transpeptidase, ALP: alkaline phosphatase, CEA: carcinoembryonic antigen, CA 19-9: carbohydrate-Antigen 19-9, AFP: Alpha-fetoprotein.

## Discussion

Despite being the second most common primary malignant tumor of the liver, CCA represents a rare type of cancer compared to other malignancies^37^. Therefore, especially due to small cohort sizes, data on diagnostic or prognostic serum based biomarkers are scarce^38,39^. Despite the existence of more or less standardized preoperative assessment algorithms (including imaging, liver function tests and the clinical performance status) it has remained challenging to predict which patients will actually benefit from extended liver surgery in terms of postoperative survival. At present, CA19-9 represents the clinically most established biomarker for CCA^24^, but its potential for the detection of CCA and its capabilities to predict patients’ outcome is still controversial^16,21,29^. Here, we showed in large cohort of CCA patients undergoing tumor resection at a tertiary referral centre that preoperative levels of circulating CEA but not CA19-9 are an independent prognostic factor and its diagnostic value for early detection of CCA might even be superior to CA19-9 in the setting of a pre-existing benign cholangiopathy like PSC.

Even in rare tumor type like CCA, it has been well established that certain clinical and pathological parameters have an important impact on the patients’ prognosis following tumor resection. As such, negative histologic margins, concomitant partial hepatectomy and a well-differentiated tumor were previously demonstrated as prognostic factors in patients with hilar cholangiocarcinoma^40^. In line, we demonstrated that the T- and M-stages were independent prognostic factors for resected patients, but this analysis was based on the postoperative histopathological assessment. However, most of these parameters, such as negative resection margins or the exact T-status, are not available in the preoperative decision making, highlighting the need for standardized parameters which are accessible before surgery. CA19-9 is the most commonly used tumor marker in the setting of CCA and e.g. routinely used in the diagnosis of the disease or the monitoring of chemotherapy responses^15,19,27,35^. It was also suggested to have a prognostic implication for CCA patients undergoing tumor resection^17,18^. However, while preoperative CA19-9 levels using a cut-off value of 324.15 U/ml discriminated between survivors and non-survivors in Kaplan-Meier curve analysis (see Fig. 3a), statistical significance for the prediction of long-term survival was not reached in univariate Cox-regression analysis in our cohort of patients (see Table 3). Furthermore, CRP levels were recently suggested as a prognostic factor in patients undergoing tumor resection for intrahepatic CCA^25^. In line, we also found in our cohort of patients that preoperative CRP serum levels are a predictive factor for long-term survival in Kaplan Meier curve analysis and univariate Cox-regression analysis. However, CRP serum levels were higher in patients with advanced TNM-stage (see Suppl. Figure 1c, g and k) which likely reflected an increased rate of cholangitis in e.g. locally advanced tumours. Hence, multivariate Cox-regression analysis including these parameters did not reveal CRP levels as an independent prognostic factor (see Table 3).

The most important finding of our study was that in contrast to CA19-9 and CRP, CEA serum levels at a cut off-value of 4.55 µg/l not only differentiated well between survivors and non-survivors but stood out as the only serum based independent prognostic factor in both univariate and multivariate Cox-regression analysis (see Table 3). CEA is a glycoprotein involved in cell adhesion processes during the fetal development of gastrointestinal tissue^41^. Usually, the production of CEA ends shortly before birth, but elevations of CEA serum levels can be observed in patients with various malignancies^14,22^. As such, CEA is routinely used as a tumor marker in the diagnostic workup and the surveillance of patients with colorectal carcinoma^22,23^. On the histological level, an upregulated expression of CEA was also shown in CCA tumor tissue^42^. Interestingly, well-differentiated CCA tumours expressed CEA primarily at the apical surface of the tumor glands, whereas the more dedifferentiated tumours showed an increased expression not only at the apical but also at the basolateral surface and in the cytoplasm^43^. In line, we found a strong trend (p = 0.066) towards higher serum levels of CEA in patients with poorly differentiated tumours compared to moderately differentiated tumor (see Suppl. Figure 2f), arguing that serum CEA can give additional information about tumor biology. However, in the setting of CCA, circulating levels of CEA are less frequently available than CA19-9 levels in daily routine and are often not taken into consideration for treatment decisions in the interdisciplinary tumor board. Here, we showed that CEA serum levels represent an independent prognostic factor for patients undergoing extended tumor resection and should probably be considered as a standard biomarker in the preoperative stratification process for patients with CCA. Importantly, our analysis only gave information on the prognosis of these patients but had no predictive value, meaning that it is unclear if those patients with an unfavourable prognosis in terms of their CEA serum levels might have had benefited to a greater extent from other therapies. However, as prognostic risk scores for CCA are currently not well established^44^, serum biomarkers could represent a valuable addition to the existing preoperative assessment algorithms to find an ideal and individual therapeutic approach for CCA patients who are often only stratified on imaging and clinical performance scores. In this setting, it is likely that especially preoperative CEA serum concentrations might be integrated into predictive algorithms as a surrogate for different aspects regarding the CCA pathophysiology, rather than being used as “stand alone” marker which determines the patients´ treatment.

Based on the standard CEA cut-off value of 5 µg/l at our institution, only a minority of CCA patients in our cohort (22.2%) showed a “positive” test result. Accordingly, the ideal CEA cut-off value that we established for our cohort for the differentiation between CCA patients and healthy controls (1.55 µg/l) was distinctly lower than the standard cut-off value and even when comparing CCA patients with our smaller cohort of PSC patients this respective cut-off value was only slightly higher (1.85 µg/l), suggesting a low variation of CEA levels in a setting of chronic cholangitis. In contrast, the cut-off values of CA19-9 differed more substantially when comparing CCA patients to healthy controls (15.75 U/ml) versus PSC patients (78.9 U/ml), corroborating the clinical notion that CA19-9 is not specific enough for the surveillance of PSC patients because CA19-9 is often elevated in cholangitis^45^. In line, in the subgroup of PSC patients analysed in this study, CA19-9 but not CEA significantly correlated with CRP (Suppl. Fig. 4a and b). Moreover, patients with common bile duct stones (CBD stones) had similar circulating levels of CEA but significantly elevated levels of CA19-9 compared to healthy controls, further arguing that CEA might be the more specific biomarker in the setting of CCA (Suppl. Fig. 4c and d). As another limitation of CA19-9, it should also be kept in mind that patients with Lewis negative blood type are unable to express CA19-9 and might therefore be diagnosed as “false-negative”. Despite the interesting hypothesis raised in our study, the exact value of CEA serum levels in the surveillance of PSC patients and the often difficult diagnosis of CCA in this setting is presently not clear and would warrant prospective analysis in a larger cohort of PSC patients. However, given that the diagnostic and prognostic cut-off values in our cohort lay below the laboratory standard cut-off value of 5 µg/l, our present data suggest that beyond the information on whether a patient is “positive” or “negative” for CEA, the exact values for CEA even below the standard cut-off value can have an important diagnostic and prognostic impact and should therefore be recognized in daily clinical care.

## Patients and Methods

### Study design and patient characteristics

This observational cohort study was designed to evaluate CA19-9, CEA, CRP and other standard laboratory parameters as diagnostic or prognostic serum markers in patients with resectable cholangiocarcinoma. Patients were recruited at University Hospital RWTH Aachen between 2010 and 2016. A total of 190 patients that were admitted for surgery of cholangiocarcinoma (see Table 1) included into this study. Serum samples were collected before any treatment prior to surgery. As a control group, 50 healthy blood donors who are medically examined on a regular basis and showed no sign of hepatic benign or malignant disease were included. Furthermore, 24 patients treated for primary sclerosing cholangitis (PSC) in our outpatient clinic who showed no evidence of malignant transformation were included as a reference group with benign hepatobiliary disease. All PSC patients had been enrolled in an intensive surveillance protocol, including regular clinical and laboratory testing (CA19-9, CEA, AST, ALT, ALP, GGT) as well as regular abdominal ultrasound and CT/MRI-scans. Moreover, we included a cohort of 40 patients with common bile duct stone as a further control population. The study protocol was approved by the local ethics committee and conducted in accordance with the ethical standards laid down in the Declaration of Helsinki and in accordance with the institutional standards (ethics committee of the University Hospital RWTH Aachen, RWTH University, Aachen, Germany).

### Measurement of laboratory parameters

All laboratory markers were measured in the local laboratory (Labordiagnostisches Zentrum, LDZ) at University Hospital RWTH Aachen. Circulating levels of serum tumor markers were analyzed with an electrochemiluminescence immunoassay (ECLIA) using the Cobas 8000 e602 modular analyzer series (Hoffmann-La Roche AG, Basel, Switzerland). Standard hematological and clinical chemistry parameters were measured using the Sysmex XN9000 (Sysmex GmbH, Norderstedt, Germany) and the Cobas 8000 c701 (Hoffmann-La Roche AG, Basel, Switzerland).

### Statistical analysis

Serum data are given as median and range to reflect the skewed distribution of analysis on human samples. Statistical analyses were performed as recently described^46^. In brief, Kolmogorov-Smirnov- and Shapiro-Wilk-Test were used to test for normal distribution. Non-parametric data were compared using the Mann-Whitney-U-Test and for multiple comparisons the Kruskal-Wallis-Test. Box plot graphics display a statistical summary of the median, quartiles and ranges. Correlation analyses were performed using the Spearman correlation tests. ROC curves were generated by plotting sensitivity against 1-specificity. The optimal cut-off values for ROC curves were established using the Youden-Index (YI = sensitivity + specificity −1). Kaplan-Meier curves were plotted to display the impact on survival. Log-rank test was used to test for differences between subgroups in Kaplan-Meier curve analysis. The prognostic value of the variables was further tested by univariate and multivariate analysis in the Cox regression model. All statistical analyses were performed with SPSS 23 (SPSS, Chicago, IL, USA)^47^. A p-value of < 0.05 was considered statistically significant (*p < 0.05; **p < 0.01; ***p < 0.001)

### Data availability

The datasets generated and analysed during the current study are available from the corresponding author on reasonable request.

## Electronic supplementary material

Supplementary Information
