# Supplementary material for: CEA but not CA19-9 is an independent prognostic factor in patients undergoing resection of cholangiocarcinoma
Source: Sci Rep. 2017 Dec 5;7:16975. doi: 10.1038/s41598-017-17175-7 (PMC5717041; doi:10.1038/s41598-017-17175-7)
Supplement: Supplementary file 1 — Supplementary Information [file 41598_2017_17175_MOESM1_ESM.pdf]

CEA but not CA19-9 is an independent prognostic factor in patients undergoing resection of cholangiocarcinoma

Sven H. Loosen[1,*], Christoph Roderburg[1,*], Katja L. Kauertz[1], Alexander Koch[1], Mihael Vucur[2], Anne T. Schneider[2], Marcel Binnebösel[3], Tom F. Ulmer[3], Georg Lurje[3], Wenzel Schoening[3], Frank Tacke[1], Christian Trautwein[1], Thomas Longerich[4], Cornelis H. Dejong[3,5], Ulf P. Neumann[3,5#], Tom Luedde[1,2,#]

[1] Department of Medicine III, University Hospital RWTH Aachen, Pauwelsstrasse 30, 52074 Aachen, Germany
[2] Division of Gastroenterology, Hepatology and Hepatobiliary Oncology, University Hospital RWTH Aachen, Pauwelsstrasse 30, 52074 Aachen, Germany
[3] Department of Visceral and Transplantation Surgery, University Hospital RWTH Aachen, Pauwelsstrasse 30, 52074 Aachen Germany
[4] Institute of Pathology, University Hospital RWTH Aachen, Pauwelsstrasse 30, 52074 Aachen, Germany
[5] Department of Surgery, Maastricht University Medical Centre (MUMC), PO Box 5800, Maastricht, The Netherlands

[*] These authors share first authorship
[#] These authors share senior authorship

Supplementary Data

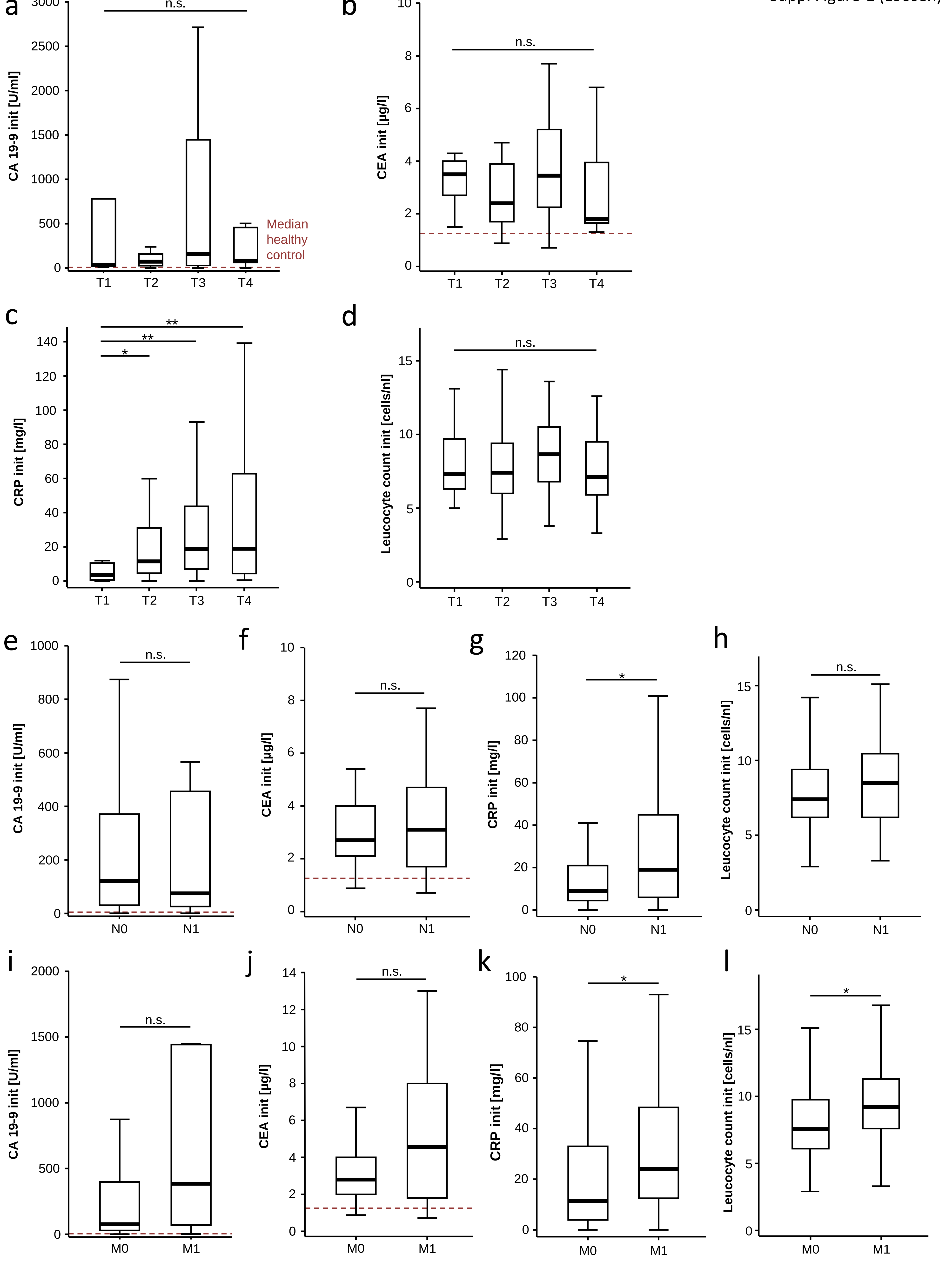
a
CA 19-9 init [U/ml]
n.s.
Median healthy control
T1 T2 T3 T4
b
CEA init [µg/l]
n.s.
c
CRP init [mg/l]
*
**
**
d
Leucocyte count init [cells/nl]
n.s.
e
f
g
h
N0 N1
i
j
k
l
M0 M1

Supplementary Figure 1

Circulating levels of CA19-9 and CEA as well as the leucocyte count are unaltered between different T-stages (a, b and d, the red dashed line reflects the median healthy control value) but patients with advanced local tumor stage (T2, T3 and T4) show significantly higher CRP levels compared to T1 patients (c). CRP levels (g) but not CA19-9 (e), CEA (f) or the leucocyte count (h) are elevated in patients with node-positive disease. CCA patients with extrahepatic CCA and intrahepatic or local peritoneal tumor spreading (M1) show a non-significant trend towards higher levels of CA19-9 (i) and CEA (j) and displayed significantly elevated levels of CRP (k) and the leucocyte count (l).

Supp. Figure-2 (Loosen)

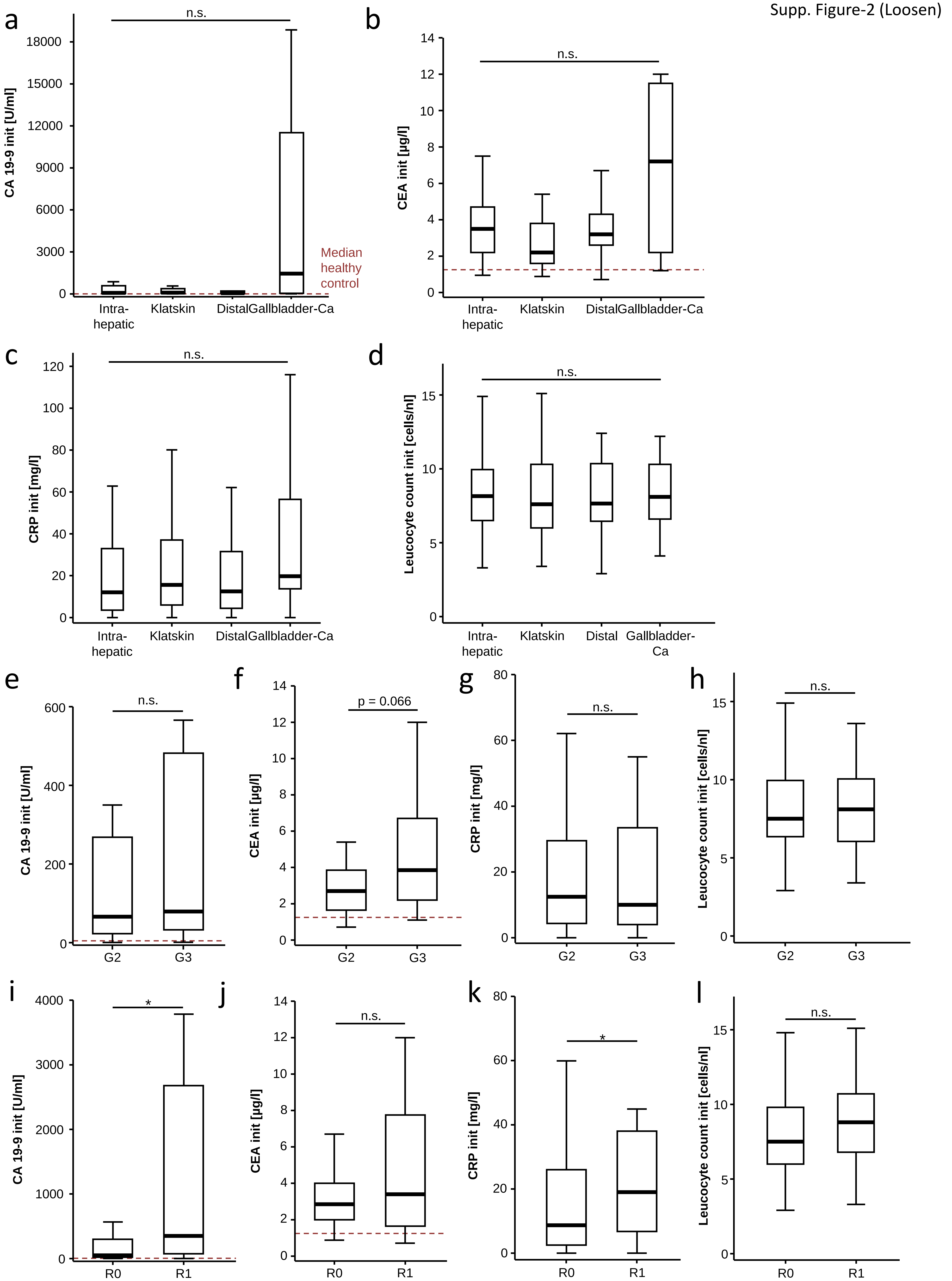

Supplementary Figure 2

There are no significant alterations regarding the CEA, CA19-9 and CRP levels as well as the leucocyte count when comparing different anatomical tumor localizations (a-d, the red dashed line reflects the median healthy control value). Poorly differentiated tumors (G3) show a strong trend (p=0.066) towards higher levels of CEA compared to moderately differentiated tumours (G2, f), whereas serum levels of CA19-9, CRP and the leucocyte count are unaltered between G2 and G3 tumours (e, g and h). The subgroup of patients that where resected with positive residual tumor cell resection (R1) have elevated initial CA19-9 and CRP but not CEA serum levels nor an elevated leucocyte count (i-l).

Supp. Figure-3 (Loosen)

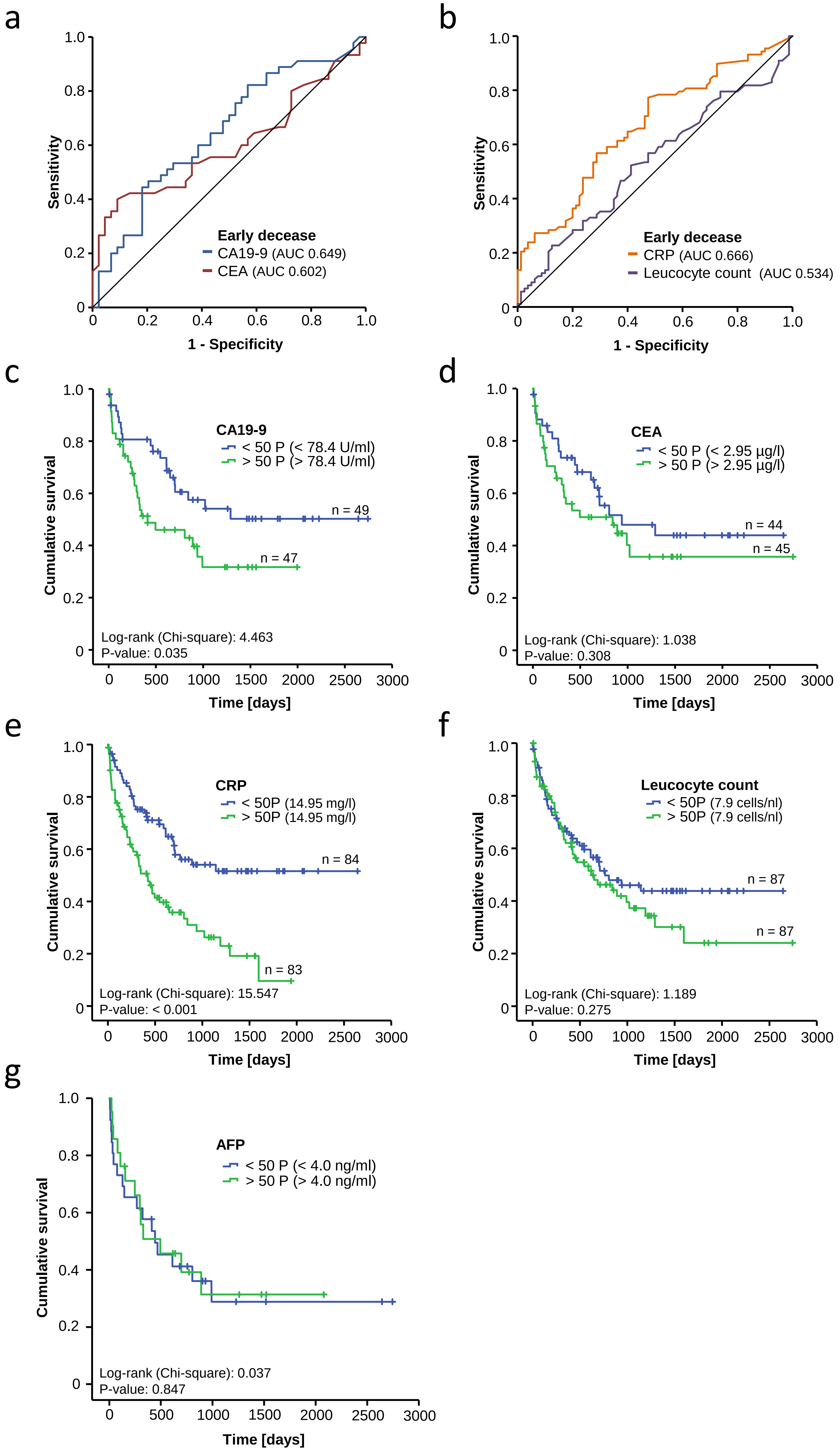

Supplementary Figure 3

ROC curve analysis reveals an AUC of 0.649 and 0.602 for CA19-9 and CEA for the prediction of early decease (a). In the same setting, CRP levels display an AUC of 0.666 while the leucocyte count had an inferior AUC of 0.534 (b). Kaplan-Meier curve analysis reveals that only CA19-9 (c) and CRP (e) but not CEA (d) and the leucocyte count (f) indicate significantly impaired long-term survival in patients with serum levels above the 50th percentile. Serum levels of AFP are unsuitable for the prediction of long term survival (g)

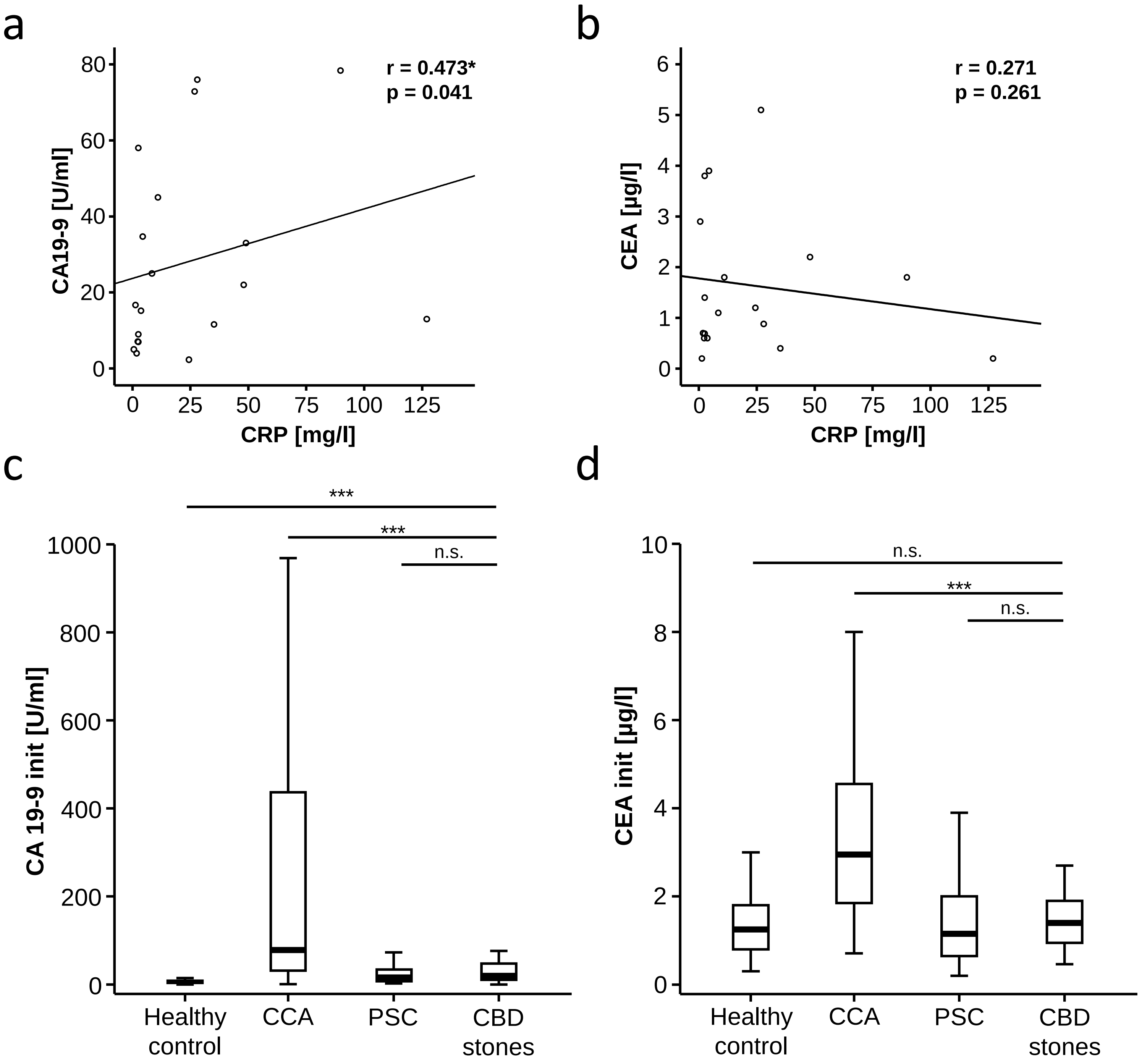

Supplementary Figure 4
CA19-9 (a) but not CEA (b) serum levels in PSC patients significantly correlate with CRP levels. Patients with common bile duct stones (CBD stones) have similar circulating levels of CEA (d) but significantly elevated levels of CA19-9 (c) in comparison to healthy controls.
